# Community Composition and Spatial Distribution of N-Removing Microorganisms Optimized by Fe-Modified Biochar in a Constructed Wetland

**DOI:** 10.3390/ijerph18062938

**Published:** 2021-03-13

**Authors:** Wen Jia, Liuyan Yang

**Affiliations:** State Key Laboratory of Pollution Control and Resource Reuse, School of the Environment, Nanjing University, Nanjing 210023, China; amethyst_wen@126.com

**Keywords:** Fe-modified biochar, constructed wetland, denitrification, anammox, denitrifier community

## Abstract

Microbial nitrogen (N) removal capability can be significantly enhanced in a horizontal subsurface flow constructed wetland (HSCW) amended by Fe-modified biochar (FeB). To further explore the microbiological mechanism of FeB enhancing N removal, *nirS*- and *nirK*-denitrifier community diversities, as well as spatial distributions of denitrifiers and anaerobic ammonium oxidation (anammox) bacteria, were investigated in HSCWs (C-HSCW: without biochar and FeB; B-HSCW: amended by biochar; FeB-HSCW: amended by FeB) treating tailwater from a wastewater treatment plant, with C-HSCW without biochar and FeB and B-HSCW amended by biochar as control. The community structures of *nirS*- and *nirK*-denitrifiers in FeB-HSCW were significantly optimized for improved N removal compared with the two other HSCWs, although no significant differences in their richness and diversity were detected among the HSCWs. The spatial distributions of the relative abundance of genes involved in denitrification and anammox were more heterogeneous and complex in FeB-HSCW than those in other HSCWs. More and larger high-value patches were observed in FeB-HSCW. These revealed that FeB provides more appropriate habitats for N-removing microorganisms, which can prompt the bacteria to use the habitats more differentially, without competitive exclusion. Overall, the Fe-modified biochar enhancement of the microbial N-removal capability of HSCWs was a result of optimized microbial community structures, higher functional gene abundance, and improved spatial distribution of N-removing microorganisms.

## 1. Introduction

The uncontrolled discharge of nitrate (NO_3_^−^-N) in water has triggered a series of serious environmental problems and health threats [1,2]. The dominant nitrogen (N) form in the tailwater from wastewater treatment plants (WWTPs) is NO_3_^−^-N, a primary cause of increased N levels in receptor waterbodies [3]. Compared with traditional methods, constructed wetlands (CWs) are highly economical and efficient in removing nutrients, organics, and heavy metals from polluted water (especially from tailwater from WWTPs) [4,5]. Biochar, as a carbon-rich material, is produced by pyrolyzed plants and animals at high temperatures with limited O_2_ supply [6]. Due to its strong physical stability, high micropore volume, and specific surface area, biochar has been used successfully to improve pollutant removal in CWs by enhancing pollutant adsorption and biofilm formation [7,8]. The functions of biochar can be further enhanced after modification [9]. Notably, iron (Fe), as a reactive element, plays a critical role in N biochemical cycles [10]. N-removal processes such as nitrification, denitrification, and anaerobic ammonium oxidation (anammox) can be facilitated by different valence and chemical forms of Fe, increasing the wastewater N-removal rate [11,12,13]. Previous studies have shown that Fe-modified biochar can increase microbial abundance and enhance N removal (especially for NO_3_^−^-N) in various wastewater treatment systems [14,15,16]. Li et al. [17] found that FeCl_3_-modified biochar could significantly enhance NO_3_^−^-N removal from water because of an increased adsorption capacity. Thus, the Fe-modified biochar could act as a promising amendment to CWs to further reduce the N concentration of wastewater (especially NO_3_^—^N-dominant wastewaters). However, information on the mechanism of N removal enhanced by Fe-modified biochar in CWs is still lacking.

N transformation and removal pathways in CWs mainly include plant uptake, substrate adsorption, and microbial activities [18]. Among them, microorganisms are considered to be major contributors to N transformation and removal [19,20]. In a variety of classical and newly discovered microbial processes, denitrification and anammox are the only two permanent N-removal pathways. The denitrification process is mediated by a large number of phylogenetically unrelated physiological groups [21]. Previous studies have shown that the abundance and composition of the CW denitrifier community can be influenced by environmental factors, including substrate type, dissolved organic carbon content, and NO_3_^−^-N level [22,23,24,25]. Thus, comprehensively investigating the associations between environmental factors and denitrifier abundance/composition is incredibly important. However, information on the spatial distribution of denitrifier community abundance and composition is rather limited in CWs. Moreover, little is known about the influence that environmental factors have on the spatial distribution of anammox bacteria in CWs, although this ecosystem type is extremely conducive to anammox [26].

Previous studies on the composition and diversity of CW microorganisms (especially denitrifiers) were mainly based on traditional molecular biology approaches, such as terminal restriction fragment length polymorphism (TRFLP) [27], clone library analysis [28,29], and denaturing gradient gel electrophoresis (DGGE) [30]. These approaches can cause an underestimation of microbial diversity and diminish our understanding of functional microbial communities. Fortunately, a high-throughput sequencing (HTS) approach can provide a more accurate analysis of the denitrifier community in complex ecosystems. To date, this analysis has been widely used to investigate denitrifier communities in various ecosystems, such as lake water and sediment, soil, and river periphytic biofilms [31,32,33,34]. However, there is still a lack of studies on applying the HTS approach to investigate the denitrifier community in CWs. Furthermore, *nirS* and *nirK* genes are the two commonly used molecular markers to detect the denitrifier community in ecosystems [28,35]. In our previous study, we indicated that FeCl_3_-modified biochar (FeB) was an effective amendment to horizontal subsurface flow constructed wetlands (HSCWs); it can enhance their microbial N-removal capability, resulting in a significant reduction of N concentration in WWTP effluent. The abundance of functional genes involved in denitrification and anammox significantly increased in FeB-HSCW [36]. However, the effect of FeB on the spatial distribution and community composition of microorganisms involved in denitrification and anammox remains unclear.

In this study, we investigate the effect of FeB on the spatial distribution and community composition of N-removing microorganisms in CWs. The overall aim is to further explore the microbiological mechanism of enhanced N removal, influenced by FeB, in CWs. The two primary objectives of this study are (1) to investigate the compositions and diversities of *nirS*- and *nirK-* denitrifier communities using Illumina MiSeq HTS in order to gain a broader understanding of FeB influence on denitrifier communities; (2) to determine the relative abundance of genes involved in denitrification and anammox by quantitative polymerase chain reaction (q-PCR), and analyze the influence of FeB on the spatial distributions of denitrifier and anammox bacteria in FeB-HSCW.

## 2. Materials and Methods

### 2.1. The Preparations and Characteristics of Biochar and Fe-Modified Biochar

Biochar was derived from bamboo (Hangzhou Linan Yaoshi Charcoal Industry Co. Ltd., Hangzhou, Zhejiang, China) by pyrolysis at 600 °C. Its average diameter was 5 mm, its bulk density was 0.75 g/cm^3^, and the specific surface area was 2.5 × 108 m^2^/m^3^. To make more activated sites available, biochar was immersed in HCl solution for 2 h, then washed repeatedly with distilled water [37]. A part of washed biochar was then immersed in FeCl_3_ solution for 2 h and also washed repeatedly to obtain Fe-modified biochar. Finally, the biochar and Fe-modified biochar were dried to a constant weight. More details on the preparations and characteristics of biochar and Fe-modified biochar are described in our previous study [36].

### 2.2. Construction and Operation of HSCWs

**Figure 1:**
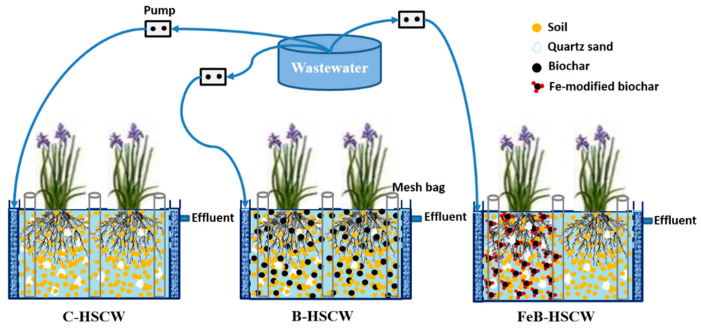
The schematic diagram of constructed wetlands (HSCWs) with different substrates. C-HSCW, without biochar and FeB-modified biochar; B-HSCW, with biochar; FeB-HSCW, with FeB-modified biochar.

Three HSCW mesocosms (working zone: 160 cm length × 30 cm width × 60 cm depth) were successfully built indoors, with *Iris hexagonus* (density: 13 plants/m^2^) planted on their surfaces (Figure 1). Each system was filled with different substrates. The control mesocosm (C-HSCW) was packed with soil and quartz sand (1:1, *w*/*w*); the biochar mesocosm (B-HSCW) was packed with soil, quartz sand, and 10% (*w*/*w*) unmodified biochar; FeB-HSCW was packed with soil, quartz sand, and 10% (*w*/*w*) Fe-modified biochar. Considering dissimilatory nitrate reduction to ammonium (DNRA), Fe-modified biochar was only added into the front half of the system [38]. To facilitate the collection of microbial samples, several mesh bags (3 cm in diameter, 60 cm in height, with a 0.1-cm mesh), filled with the corresponding substrate, were evenly embedded in each HSCW during the construction of systems.

Before starting this study, the HSCWs were operated consistently for 8 months under different combinations of influent N loading and hydraulic retention time (HRT) to purify the effluent from Wunan WWTP (Changzhou, Jiangsu, China). The influent characteristics of HSCWs in each operated stage are summarized in Table S1. FeB-HSCW exhibited significantly more effective N-removal capability (Table S2), and its highest removal efficiency was approximately 6.0- and 2.0-fold higher than those of C-HSCW and B-HSCW, respectively. More details on the construction and operation of the HSCWs can be found in our previous study [36].

### 2.3. Microorganism Sample Collection and DNA Extration

Mesh bags, in triplicate, were collected from six equidistant points in each HSCW. The microorganism samples in each bag were divided into four equal portions based on the vertical depth (0–15, 15–30, 30–45, and 45–60 cm). The total genomic DNA of each sample (0.5 g) was extracted with the Fast DNA^®^ SPIN Kit (MP Biomedicals, Santa Ana, CA, USA). Its quantity and quality were also determined before the follow-up experiments.

### 2.4. High-Throughput Sequencing of nirS and nirK Genes

For the denitrifier community analysis, all microbial samples obtained from the same HCSW were combined into one sample. Each mixed sample (0.5 g), in duplicate, was used to extract the total genomic DNA, and the quality and quantity of the extracted DNA were subsequently evaluated. The *nirS* and *nirK* genes were amplified for HTS analysis by using the primers cd3aF/R3cd and F1aCu/R3Cu, respectively [35]. PCR products were purified and pooled in equimolar amounts and were then measured on a MiSeq Illumia platform (Majorbio, Shanghai, China). More information on sequence processing is described in previous work [35,39]. Using UPARSE (version 7.1, http://drive5.com/uparse/ (15 December, 2020)), sequences were clustered into operational taxonomic units (OTUs) at a sequence identity of 97%. At a 3% distance level, ACE richness, Sobs richness, Chao richness, Shannon diversity, and Simpson diversity were calculated and analyzed through the use of Mothur (http://www.mothur.org (15 December, 2020)), based on the OTUs. The sequences were grouped into different taxonomy levels using the Ribosomal Database Project (RDP) Classifier (version 2.2, http://sourceforge.net/projects/rdp-classifier/ (15 December, 2020)) via the functional gene database (http://fungene.cme.msu.edu/ (15 December, 2020)), with a threshold of 70%.

### 2.5. Quantitative Analysis of Genes Involved in Denitrification and Anammox

A quantitative analysis of the genes mediating denitrification and anammox was performed using q-PCR assays, including the bacterial *16S* rRNA gene, the anammox bacterial *16S* rRNA gene (*amx*), and functional genes *narG*, *napA*, *nirS*, *nirK*, *qnorB*, *cnorB*, *nosZ*-I, *nosZ*-II, and *hzsA*. More information on the primers is summarized in Table S3, and the protocols and parameters used for each gene amplification are listed in Table S4. Each amplifying reaction was performed in triplicate. More details of the qPCR operations are described in our previous study [36].

### 2.6. Statistical Analysis

Data analysis and visualization were performed using Origin 9.0 software. All data are presented as mean ± SD. Statistical checks were performed at a significance level of 0.05 using one-way ANOVA in SPSS 17.0 (SPSS Inc., Chicago, IL, USA). The differences in denitrifier community richness and diversity were tested using Student’s *t*-test. To compare the differences in *nirS*- and *nirK*-denitrifier community compositions among HSCWs, a weighted UniFrac distance was obtained and then hierarchical clustering was carried out based on the weighted pair group method using R (version i386, 3.3.0) (John Fox, Hamilton, Canada). The abundances of functional genes were analyzed as relative abundances (gene copy numbers/bacterial *16S* rRNA gene for *narG*, *napA*, *nirS*, *nirK*, *qnorB*, *cnorB*, *nosZ*-I, and *nosZ*-II; gene copy numbers/*amx* for *hzsA*).

## 3. Results

### 3.1. The Richness and Diversity of Denitrifiers in the Three HSCWs

Here, the HTS approach was applied to estimate the effects of Fe-modified biochar on *nirS*- and *nirK*-denitrifier richness and diversity in HSCWs treating the effluent from WWTPs (Table 1). In total, 94,707 and 101,452 valid reads of the *nirS* and *nirK* genes, respectively, were retrieved from the MiSeq Illumina sequencing platform. The average lengths of these two genes were ~392 and ~452 bp, respectively. Good’s coverage estimator (~ 99%) suggested that the OTUs of the *nirS* and *nirK* gene libraries in each HSCW were well-captured. The OTU numbers of *nirS* and *nirK* genes in FeB-HSCW were 795 and 802, respectively, which were both significantly higher than those in C-HSCW and B-HSCW. The richness and diversity of *nirS*- and *nirK*-denitrifiers varied among the HSCWs. In FeB-HSCW, the richness and diversity indices of Sobs, Chao 1, ACE, Shannon, and Simpson were 451, 523.60, 523.76, 4.68, and 0.022, respectively, for the *nirS* gene and 444.5, 480.05, 487.15, 4.80, and 0.019, respectively, for the *nirK* gene. Moreover, no significant differences (*p* > 0.05) in these indices, among the HSCWs, were detected by Student’s *t*-test.

### 3.2. The Community Structures of Denitrifiers in the Three HSCWs

**Figure 2:**
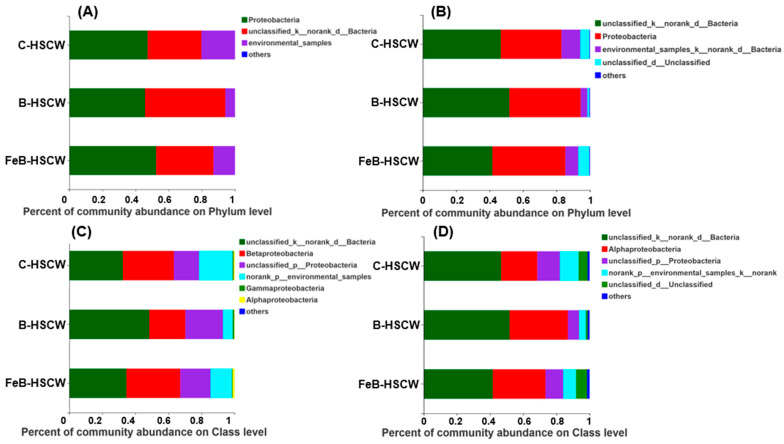
The community structures of *nirS*-denitrifiers at the phylum level (**A**), *nirK*-denitrifiers at the phylum level (**B**), *nirS*-denitrifiers at the class level (**C**), and *nirK*-denitrifiers at the class level (**D**) in the three constructed wetlands (HSCWs). C-HSCW, without biochar and FeB-modified biochar; B-HSCW, with biochar; FeB-HSCW, with FeB-modified biochar.

Proteobacteria and unclassified_k_norank_d_Bacteria were the dominant groups for both *nirS*- and *nirK*-denitrifiers in the three HSCWs at the phylum level (Figure 2A,B). Notably, the relative proportions of Proteobacteria for these two types of denitrifiers in FeB-HSCW were 52.42% and 43.59%, respectively, which were higher than those in C-HSCW and B-HSCW. The relative proportion of unclassified_k_norank_d_Bacteria ranged from 32.44% to 51.80%, followed by B-HSCW > FeB-HSCW > C-HSCW for *nirS*-denitrifiers and B-HSCW > C-HSCW > FeB-HSCW for *nirK*-denitrifiers. Compared with the other two HSCWs, there was another group of *nirS*-denitrifiers in FeB-HSCW (sequences not classified into any known group). For the *nirK*-denitrifiers, environmental_samples_k_norank_d_Bacteria (4.06–11.27%) and unclassified_d_Unclassified (1.16–6.63%) were the subdominant groups, but their relative proportions varied among the three systems.

At the class level (Figure 2C,D), unclassified_k_norank_d_Bacteria was the dominant group for both *nirS*- and *nirK*-denitrifiers in the three HSCWs, and their relative proportions (*nirS*-: 34.57%; *nirK*-: 41.66%) were lower in FeB-HSCW than those in B-HSCW (*nirS*-: 48.38%; *nirK*-: 51.80%). β-Proteobacteria was another dominant group for *nirS*-denitrifiers, and the highest relative proportion was detected in FeB-HSCW (32.55%). In addition, α-Proteobacteria were more abundant in FeB-HSCW (1.16%) than in the other two HSCWs, whereas γ-Proteobacteria content was relatively low (0.44%). In contrast to *nirS*-denitrifiers, α-Proteobacteria was the subdominant group for *nirK*-denitrifiers in HSCWs, with a relatively high abundance observed in FeB-HSCW (31.77%).

**Figure 3:**
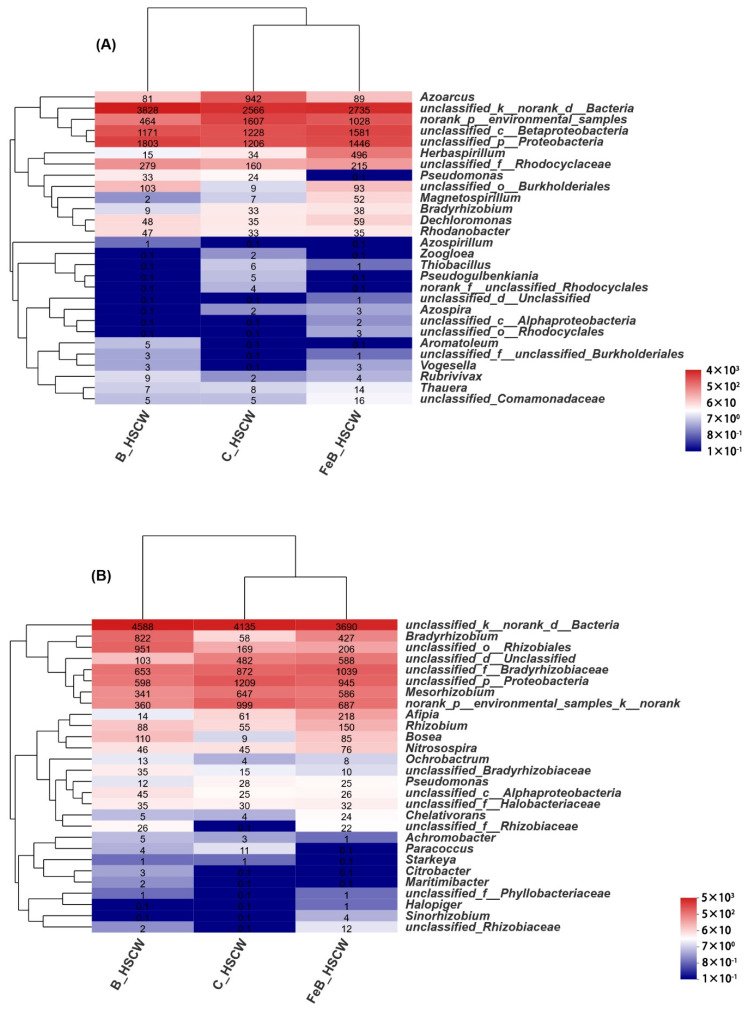
The community structures of *nirS*-denitrifiers (**A**) and *nirK*-denitrifiers (**B**) at the genera level (**A**) in the three constructed wetlands (HSCWs; heat map of hierarchy cluster for the top 50 genera). C-HSCW, without biochar and FeB-modified biochar; B-HSCW, with biochar; FeB-HSCW, with FeB-modified biochar.

To obtain more comprehensive insights into similarities and differences in the community structures for *nirS*- and *nirK*-denitrifiers, a heat map of hierarchical clustering for the 50 most abundant genera is shown in Figure 3A,B, respectively. For *nirS*-denitrifiers, significantly higher abundances of *unclassified_c_β-Proteobacteria* (19.98%), *Herbaspirillum* (6.27%), *Dechloromonas* (0.75%), *Magnetospirillum* (0.66%), *Bradyrhizobium* (0.48%), *unclassified_Comamonadaceae* (0.20%), *Thauera* (0.18%), *Azospira* (0.04%), *unclassified_o_Rhodocyclales* (0.04%), *Vogesella* (0.04%), *unclassified_c_α-Proteobacteria* (0.03%), and *unclassified_d_Unclassified* (0.01%) were observed in FeB-HSCW compared to the other two systems. Meanwhile for *nirK*-denitrifiers, *unclassified_f_Bradyrhizobiaceae* (11.72%), *unclassified_d_Unclassified* (6.63%), *Afipia* (2.46%), *Rhizobium* (1.69%), *Nitrosospira* (0.86%), *Chelativorans* (0.27%), *unclassified_Rhizobiaceae* (0.14%), *Sinorhizobium* (0.05%), *unclassified_f_Phyllobacteriaceae* (0.01%), and *Halopiger* (0.01%) were observed.

However, the enrichment of genera *Pseudomonas* (0.001%), *Azospirillum* (0.001%), *Zoogloea* (0.001%), *Pseudogulbenkiania* (0.001%), norank_f_*unclassified_Rhodocyclales* (0.001%), and *Aromatoleum* (0.001%) were lowest for *nirS*-denitrifiers in FeB-HSCW. For *nirK*-denitrifiers, the lowest genera were *unclassified_k_norank_d_Bacteria* (41.63%), *unclassified_Bradyrhizobiaceae* (11.72%), *Achromobacter* (0.01%), *Paracoccus* (0.001%), *Starkeya* (0.001%), *Citrobacter* (0.001%), and *Maritimibacter* (0.001%).

### 3.3. The Spatial Distribution of Denitrifying Functional Genes in the Three HSCWs

To more accurately investigate the effects of FeB on the spatial distribution of denitrifier communities, the copy numbers of the bacterial *16S* rRNA gene, *narG*, *napA*, *nirS*, *nirK*, *qnorB*, *cnorB*, *nosZ*-I, and *nosZ*-II were first detected in HSCWs. Their average abundances were significantly higher in FeB-HSCW than in C-HSCW (2.30- to 27.84-fold) and B-HSCW (1.11- to 7.53-fold).

#### 3.3.1. Spatial Distributions of *narG* and *napA* in HSCWs

**Figure 4:**
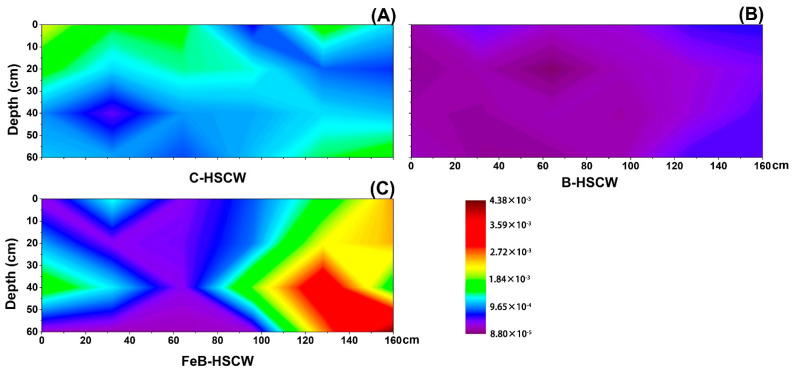
The spatial distribution of *narG* relative abundance in C-HSCW (**A**), B-HSCW (**B**), and FeB-HSCW (**C**) based on interpolated maps. Color scales indicate extrapolated values by kriging. C-HSCW, without biochar and FeB-modified biochar; B-HSCW, with biochar; FeB-HSCW, with FeB-modified biochar.

**Figure 5:**
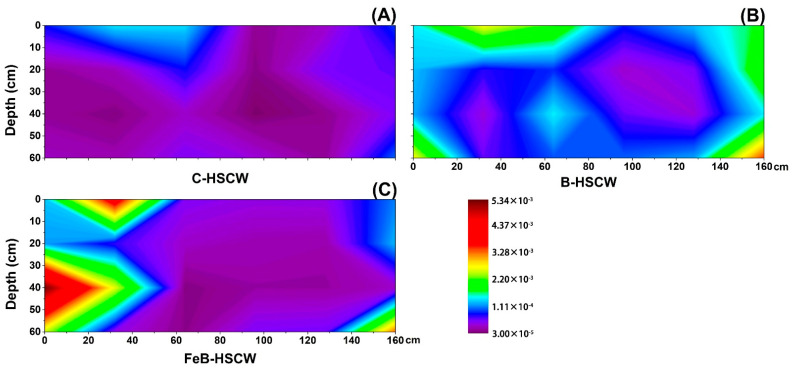
The spatial distribution of *napA* relative abundance in C-HSCW (**A**), B-HSCW (**B**), and FeB-HSCW (**C**) based on interpolated maps. Color scales indicate extrapolated values by kriging. C-HSCW, without biochar and FeB-modified biochar; B-HSCW, with biochar; FeB-HSCW, with FeB-modified biochar.

In this study, the relative abundances of *narG* and *napA* in three HSCWs treating WWTP effluent were found to vary among the sampling sites (Figure 4 and Figure 5). Compared with C-HSCW and B-HSCW, significant differences in spatial distributions of *narG* and *napA* relative abundances were found in FeB-HSCW. Higher values of *narG* and *napA* relative abundances were detected in FeB-HSCW (*narG*: 0.026–0.438%, *napA*: 0.008–0.533%). Notably, the relative abundances of *narG* and *napA* were negatively correlated in FeB-HSCW. The high values for the relative abundance of *narG* were mainly concentrated in the latter part of the system, while the *napA* was more abundant at the front part. Moreover, the relative abundance of these two genes exhibited higher value patches for *napA* and larger patches for *narG* in FeB-HSCW.

#### 3.3.2. Spatial Distributions of *nirS* and *nirK* in HSCWs

**Figure 6:**
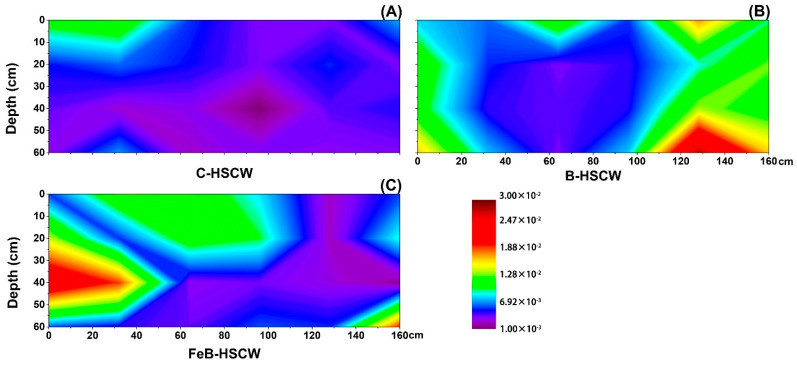
The spatial distribution of *nirS* relative abundance in C-HSCW (**A**), B-HSCW (**B**), and FeB-HSCW (**C**) based on interpolated maps. Color scales indicate extrapolated values by kriging. C-HSCW, without biochar and FeB-modified biochar; B-HSCW, with biochar; FeB-HSCW, with FeB-modified biochar.

**Figure 7:**
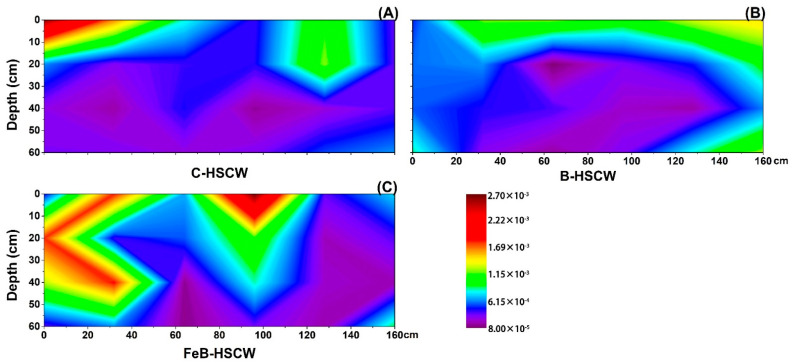
The spatial distribution of *nirK* relative abundance in C-HSCW (**A**), B-HSCW (**B**), and FeB-HSCW (**C**) based on interpolated maps. Color scales indicate extrapolated values by kriging. C-HSCW, without biochar and FeB-modified biochar; B-HSCW, with biochar; FeB-HSCW, with FeB-modified biochar.

Different spatial distributions of the relative abundance of *nirS* (Figure 6) and *nirK* genes (Figure 7) were detected in three HSCWs. The relative abundance of *nirS* and *nirK* genes in FeB-HSCW was 0.146–2.397% and 0.012–0.268%, respectively. Compared with C-HSCW and B-HSCW, more and larger high-value patches of *nirS* and *nirK* relative abundances were observed in FeB-HSCW (Figure 6C and Figure 7C). Distinct from *narG* and *napA*, similar distributions of *nirS* and *nirK* relative abundances were observed in FeB-HSCW, and the high values of both were mainly concentrated in the front and middle-upper parts. However, *nirS* and *nirK* were mainly concentrated on the surface of C-HSCW and the rear part of B-HSCW. Moreover, *nirS* was more abundant than *nirK* in FeB-HSCW, and the highest value of *nirS* relative abundance was an order of magnitude more abundant than that of *nirK*.

#### 3.3.3. Spatial Distributions of *qnorB* and *cnorB* in HSCWs

**Figure 8:**
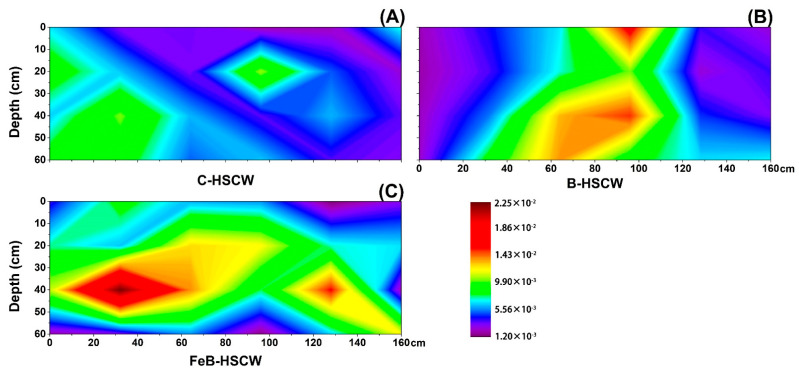
The spatial distribution of *qnorB* relative abundance in C-HSCW (**A**), B-HSCW (**B**), and FeB-HSCW (**C**) based on interpolated maps. Color scales indicate extrapolated values by kriging. C-HSCW, without biochar and FeB-modified biochar; B-HSCW, with biochar; FeB-HSCW, with FeB-modified biochar.

**Figure 9:**
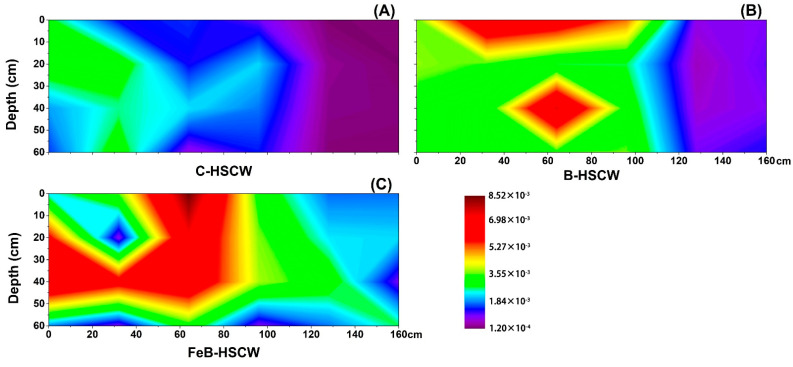
The spatial distribution of *cnorB* relative abundance in C-HSCW (**A**), B-HSCW (**B**), and FeB-HSCW (**C**) based on interpolated maps. Color scales indicate extrapolated values by kriging. C-HSCW, without biochar and FeB-modified biochar; B-HSCW, with biochar; FeB-HSCW, with FeB-modified biochar.

Among the three HSCWs, the spatial distributions of *qnorB* relative abundance were significantly different (Figure 8). *qnorB* was widely present in FeB-HSCW but was mainly present at the bottom of the front part in C-HSCW and the middle part in B-HSCW. However, the spatial distributions of *cnorB* relative abundance exhibited similar characteristics of high in the front and low at the back (Figure 9). Their relative abundances in FeB-HSCW ranged from 0.126–0.242% (*qnorB*, Figure 8C) and 0.080–0.851% (*cnorB*, Figure 9C), respectively. The patch with relatively high values in FeB-HSCW was larger than those in C-HSCW and B-HSCW, which were mainly concentrated in the first half of the system. Consistent with the *nirS*-*nirK* pair, similar distributions of *qnorB* and *cnorB* relative abundances were also found in FeB-HSCW, where *qnorB* was an order of magnitude more abundant than *cnorB*.

#### 3.3.4. Spatial Distributions of *nosZ*-I and *nosZ*-II in HSCWs

**Figure 10:**
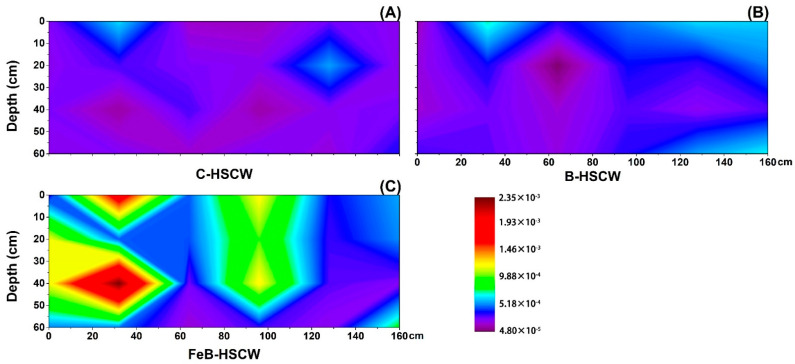
The spatial distribution of *nosZ*-I relative abundance in C-HSCW (**A**), B-HSCW (**B**), and FeB-HSCW (**C**) based on interpolated maps. Color scales indicate extrapolated values by kriging. C-HSCW, without biochar and FeB-modified biochar; B-HSCW, with biochar; FeB-HSCW, with FeB-modified biochar.

**Figure 11:**
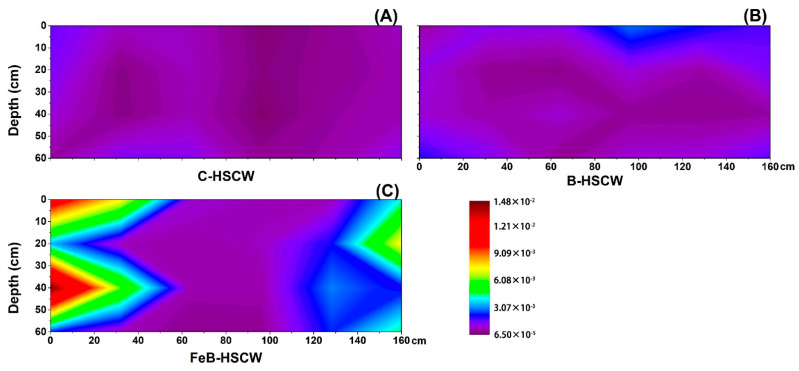
The spatial distribution of *nosZ*-II relative abundance in C-HSCW (**A**), B-HSCW (**B**), and FeB-HSCW (**C**) based on interpolated maps. Color scales indicate extrapolated values by kriging. C-HSCW, without biochar and FeB-modified biochar; B-HSCW, with biochar; FeB-HSCW, with FeB-modified biochar.

The spatial distributions of *nosZ*-I and *nosZ*-II relative abundances were more heterogeneous and complex in FeB-HSCW than in C-HSCW and B-HSCW (Figure 10 and Figure 11). Higher-value patches of *nosZ*-I relative abundance (>0.07%, Figure 10C) and *nosZ*-II relative abundance (>0.29%, Figure 11C) were only detected in FeB-HSCW by the interpolated maps. Moreover, higher-value patches of *nosZ*-I relative abundance were mainly concentrated in the front and center parts of FeB-HSCW, while the higher-value patches of *nosZ*-II relative abundance were observed in the front and back ends. Similar to the gene pairs of *nirS*-*nirK* and *qnorB*-*cnorB*, the relative abundance of *nosZ*-II was generally higher (by an order of magnitude) than that of *nosZ*-I.

### 3.4. Spatial Distribution of Anammox Genes in HSCWs

**Figure 12:**
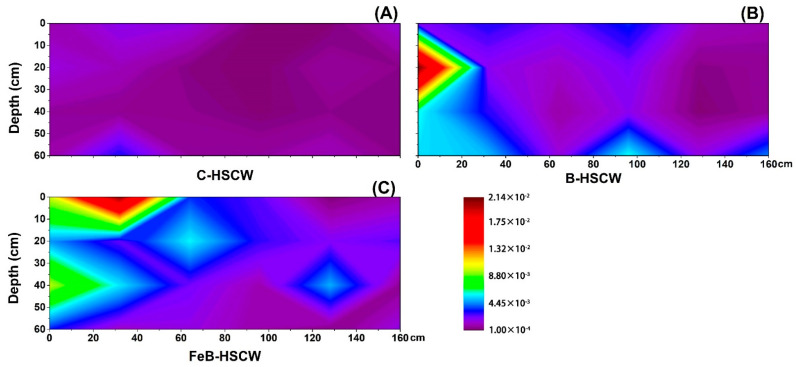
The spatial distribution of *hzsA* relative abundance in C-HSCW (**A**), B-HSCW (**B**), and FeB-HSCW (**C**) based on interpolated maps. Color scales indicate extrapolated values by kriging. C-HSCW, without biochar and FeB-modified biochar; B-HSCW, with biochar; FeB-HSCW, with FeB-modified biochar.

The spatial distributions of *hzsA* relative abundance differed significantly among the HSCWs (Figure 12). A more heterogeneous and complex spatial distribution of *hzsA* relative abundance was detected in FeB-HSCW, with the high-value patches mainly existing at the front half of the system. However, the high-value patches were only observed at the bottom of the front part in C-HSCW and the front end of B-HSCW. Notably, in contrast to those in C-HSCW and B-HSCW, higher *hzsA* enrichment (relative abundance ranging from 0.021% to 2.081%) was observed in the vast majority of the sites in FeB-HSCW.

## 4. Discussion

### 4.1. Effect of Fe-Modified Biochar on Denitrifier Richness and Diversity

Direct information on denitrifier richness and diversity in CWs, based on HTS analysis, is still very limited, although this method has been widely used to investigate bacterial community structure. Wu et al. [35] revealed that the distribution patterns of heterotrophic denitrifiers could be influenced by root exudates in micropolluted CWs. In this study, the effects of Fe-modified biochar on *nirS*- and *nirK*-denitrifier richness and diversity in HSCWs were estimated by the HTS approach. A relatively large amount of *nirS* and *nirK* gene OTUs in FeB-HSCW indicated that Fe-modified biochar could increase the microbial communities in HSCWs. However, no significant differences (*p* > 0.05) in the richness and diversity indices were observed among HSCWs, demonstrating that Fe-modified biochar had no significant influence on the richness and diversity of the denitrifier community. This was consistent with a previous study on biochar produced by rice straw [40].

### 4.2. Fe-Modified Biochar Optimizes Denitrifier Community Structures in FeB-SHCW

Microbial communities are the main promoter of the CW nutrient biogeochemical cycle, and their activities are crucial to the functioning of wetlands because they can determine energy flow and nutrient transformation. Several factors (i.e., nutrient levels, feeding pattern, and root exudates) have been reported to influence the CW denitrifiers’ community structure [25,35,39]. In this study, the community structures of *nirS*- and *nirK*-denitrifiers in HSCWs were altered by Fe-modified biochar, although Fe-modified biochar had no significant influence on their richness and diversity. The phyla Proteobacteria, the main denitrifying bacteria in CWs, have been observed in several studies [25,41]. Thus, the relatively high abundances of Proteobacteria for *nirS*- and *nirK*-denitrifiers in FeB-HSCW indicate that Fe-modified biochar can effectively increase the enrichment of the main denitrification actor, enhancing N removal. In addition, a more complex community structure of *nirS*-denitrifiers existed in FeB-HSCW as an unknown group that was only detected in this system. It was also revealed that Fe-modified biochar could promote the enrichment of new denitrifier phyla in CWs to contribute to excellent N removal, which requires extensive investigation in future studies.

Lu et al. [42] reported that sub-Proteobacteria (mainly α-, β- and γ-Proteobacteria) have an excellent denitrification capability to facilitate N removal in biological wastewater treatments. In this study, there were more abundant β-Proteobacteria and α-Proteobacteria for *nirS*-denitrifiers enriched in FeB-HSCW at the class level, whereas γ-Proteobacteria content was the lowest. These results suggest that Fe-modified biochar exhibits significant selective enrichment for *nirS*-denitrifiers, which is more conducive to the growth and reproduction of α- and β-Proteobacteria. Moreover, the relative abundance of α-Proteobacteria for *nirK*-denitrifiers was the highest in FeB-HSCW among HSCWs and was much higher than that for *nirS*-denitrifiers. Therefore, different community structures, between *nirS*- and *nirK*-denitrifiers, were formed in FeB-HSCW, probably resulting from the competitive exclusion between them [43].

At the genera level, nearly all of the detected microorganisms were heterotrophic bacteria; several of them have been observed in previous studies [44]. For instance, *Herbaspirillum*, *Magnetospirillum*, *Bradyrhizobium*, *Thauera*, *Azospira,* and *Vogesella* are known *nirS*-denitrifiers in micropolluted CWs, whereas *Rhizobium* and *Sinorhizobium* have been previously observed as *nirK*-denitrifiers [35]. Moreover, *Dechloromonas* is the dominant group in denitrifying reactors and anaerobic sludge [45,46,47]. This genus has been demonstrated to oxidize organic matter, with nitrate as the electron acceptor, and may prefer complete denitrification [48]. The relative abundance of *Dechloromonas* was higher in FeB-HSCW, indicating that Fe-modified biochar was more conducive to N transformation and N_2_O reduction. Moreover, *Thauera*, as a type of aerobic denitrifier, could effectively reduce the accumulation of NO_2_^−^-N and moderate N_2_O emissions [49]. Wang and Chu [50] pointed out that Comamonadaceae was the primary group for solid-phase denitrification. The *unclassified_Comamonadaceae* has been found in denitrifying reactors as well as electrolysis-augmented CWs [41,51]. *Afipia* is the dominant species responsible for autotrophic denitrification in the cathode of microbial fuel cells [52]. Thus, the many types of denitrifiers observed at higher abundances in FeB-HSCW indicate that Fe-modified biochar can significantly optimize denitrifier community construction as well as enhance the coexistence of various denitrifying pathways (i.e., aerobic and anaerobic, autotrophic, and heterotrophic) in the system. Biochar and Fe ions have been shown to alter the microbial community structure, respectively, to enhance N transformation and removal due to their unique physical and chemical properties [8,53]. Therefore, owing to its higher porous structure, larger surface area, and stronger adsorption capacity and the “iron wheel” effect between Fe^3+^ and Fe^2+^ transformation, Fe-modified biochar has an excellent capability to optimize denitrifier community structures and increase the enrichment of various denitrifiers in CWs for further enhancing N removal in wastewater [36].

Furthermore, *Pseudomonas* species are widely found in various environments, and some are capable of autotrophic and heterotrophic denitrification [54,55]. Notably, N loading might have a substantial impact on the abundance of this denitrifier type in various environments. In a bioelectrochemically assisted CW, dominated by genus *Pseudomonas*, the influent N concentration was greater than 130 mg/L, over 10 times higher than in this study [56]. He et al. [57] revealed that high N loadings (from 20 to 50 mg/L) could reduce the abundance of denitrifying bacteria *Zoogloea* in biochar-packed reactors. Thus, 6 genera for *nirS*-denitrifiers and 7 genera for *nirK*-denitrifiers in FeB-HSCW, with the lowest abundance among HSCWs, might be ascribed to the interaction between FeB and influent N loading.

### 4.3. Fe-Modified Biochar Influences Spatial Distribution of Denitrifying Functional Genes in FeB-HSCW

Several studies have demonstrated that denitrifier abundances in CW medium possess high spatial heterogeneity [23,58]. In this study, the spatial distributions of denitrifier abundances varied substantially among the HSCWs, according to the relative abundance distributions of *narG*, *napA*, *nirS*, *nirK*, *qnorB*, *cnorB*, *nosZ*-I, and *nosZ*-II. Among HSCWs, relative abundances of those denitrification genes were higher in FeB-HSCW, indicating that Fe-modified biochar could significantly increase the components of various denitrifiers in the bacterial community and was more beneficial to complete denitrification in FeB-HSCW. Notably, Fe-modified biochar had a significant impact on the spatial distribution of denitrifying functional genes in HSCWs. The spatial distributions of denitrifier abundances in FeB-HSCW were significantly different from those in C-HSCW and B-HSCW and showed a more heterogeneous and patchy pattern.

Previous studies have suggested that the microbial groups harboring different genes, implicated in similar paths of the denitrification process, have a differential utilization of the sediment habitat [58]. *narG* and *napA* genes encode membrane-bound and periplasmic enzymes, respectively, and exist in the same or different bacteria involved in the first step of denitrification (NO_3_^−^-N → NO_2_^−^-N) [59]. Thus, denitrifier groups harboring *narG* or *napA* occupy differential habitat locations in FeB-HSCW, probably reflecting past competitive exclusion between these two kinds of denitrifiers, and the Fe-modified biochar was more conducive to the enrichment of *napA*-denitrifiers. Moreover, the relative abundance of these two genes exhibited higher value patches for *napA* and larger patches for *narG* in the interpolated maps. Thus, the growth and reproduction of both *narG*- and *napA*-denitrifiers were effectively promoted in FeB-HSCW, simultaneously enhancing anaerobic and aerobic denitrification [60].

The *nirS* and *nirK* genes have been commonly used to investigate CW denitrifier communities [28,61]. They encode cytochrome *cd*_1_ and copper nitrite reductase, respectively, and are carried by different bacteria [62]. Two nitric oxide reductases (Nor), which catalyze the reduction of NO to N_2_O, are encoded by *qnorB* and *cnorB*, respectively [63]. Moreover, the *nosZ* gene often acts as a marker for complete denitrification [64]. Simultaneous analysis of both *nosZ*-I and *nosZ*-II genes are more conducive to more accurate and comprehensive investigations of N_2_O-reducing communities [65]. Distinct from *narG* and *napA*, similar distributions of the functionally redundant pairs (*nirS*–*nirK*, *qnorB*–*cnorB,* and *nosZ*-I–*nosZ*-II) were observed in FeB-HSCW, and patches with high values of those relative abundances were mainly concentrated in the front half of the system. Thus, Fe-modified biochar could significantly enhance the enrichment of denitrifiers harboring those functional genes in HSCWs and effectively reduce or even eliminate the competitive exclusion between denitrifiers with the same function by improving their differential utilization of habitats [66]. This could result from their very different relative abundance ranges, as *nirS*, *qnorB,* and *nosZ*-II were an order of magnitude more abundant than *nirK*, *cnorB,* and *nosZ*-I, respectively. Similar results for *nirS* and *nirK* genes were also found by Correa-Galeote et al. [58,67]. These further implied that the Fe-modified biochar could significantly promote the enrichment of *nirS*-, *qnorB*-, and *nosZ*-II-denitrifiers, which were extremely important for the excellent transformation and removal of N in FeB-HSCW. Moreover, the reduction of NO to N_2_O is considered the primary source of N_2_O produced in a wetland [68]. However, the poor stability of Nor, combined with the cytotoxic effects of NO, resulted in less focus on the NO reduction process [69]. Thus, Fe-modified biochar could significantly increase the diversity of NO-reducing bacteria and enhance the capability for NO reduction in FeB-HSCW with the primary contribution of *qnorB*-denitrifiers [63,70].

### 4.4. FeB-Modified Biochar Improved the Spatial Distribution of Anammox Genes in FeB-HSCW

The amx gene has been widely used as a marker for anammox bacteria [71]. The hzsA gene, encoding hydrazine synthase (a subunit of the specific anammox enzyme), is considered the most valuable molecular marker for anammox bacteria [72]. Humbert et al. [73] pointed out that the hzsA primers have an advantage over amx primers because the latter may amplify other Planctomycetes genes in addition to those of the anammox group. Therefore, the relative abundance of hzsA to amx was used in this study to more precisely investigate the spatial distribution of anammox bacteria. The spatial distribution of hzsA relative abundance in FeB-HSCW was the most heterogeneous and complex among HSCWs; this was similar to the distribution patterns of those denitrification functional genes. Higher hzsA enrichments were observed in the vast majority of the sites in this system compared to those in the other two HSCWs, suggesting that Fe-modified biochar could create more suitable microenvironments in FeB-HSCW to increase the enrichment of anammox bacteria [74]. In addition, high-value patches mainly existed at the front part of FeB-HSCW, indicating that the front part of FeB-HSCW was the main location for anammox. In general, anammox bacteria are less energetically active, with lower growth rates and biomass yields than heterotrophic denitrifiers. Considering that the front part of FeB-HSCW was also the primary habitat for most denitrifiers, Fe-modified biochar provided more diverse habitats to effectively reduce competitive exclusion between denitrifiers and anammox bacteria. The coexistence of these two microbial communities, involved in N removal, was effectively promoted in FeB-HSCW, which was more beneficial for enhancing the deep removal of N (especially for NO_3_^−^-N).

### 4.5. Strengthening Mechanism of Fe-Modified Biochar on Microbial N Removal in HSCW

Several previous studies have revealed the spatial dynamics of denitrifier abundance in natural wetlands and CWs [28,39]. However, information on the spatial distributions of denitrifiers and anammox bacteria in the new Fe-modified biochar-augmented CW, treating micropolluted effluent from WWTPs, is still lacking. Fe is one of the most abundant metal elements on Earth, and its oxidation–reduction process is very important in the biogeochemical cycle of N. The growth, reproduction, and metabolic activity of denitrifier and anammox bacteria can be promoted by Fe to achieve satisfactory N removal in various wastewater treatment systems [53,75,76,77,78]. Meanwhile, because of its large surface area and highly porous structure, biochar can provide enough habitats for microbial growth. The functional genes involved in denitrification and anammox can be efficiently enriched in biochar-amended systems [40,79,80]. In this study, Fe-modified biochar significantly increased the relative abundances of functional genes involved in denitrification and anammox, rendering their spatial distributions more heterogeneous and complex in FeB-HSCW. This can be ascribed to the following reasons: (1) Fe-modified biochar has a larger surface area and higher porous structure to provide more suitable habitats for the growth of various N-removing microorganisms in CWs, which significantly increase the abundances of genes involved in denitrification and anammox [36]. (2) Fe-modified biochar has a stronger adsorption capacity for organic matter, N, and phosphorus from wastewater via electrostatic attraction and intermolecular hydrogen bonding, with π–π bonds, which could effectively improve the “in situ enrichment” of these substances and then facilitate the microbial N-removal process [79,81,82,83]. (3) Fe-modified biochar might alter the microenvironment in CWs and create more favorable conditions for denitrification and anammox [36,74]. Meanwhile, Fe-modified biochar could also act as an “electron shuttle” to facilitate electron transfer in N-removal processes [84]. 4) The “iron wheel” effect between Fe^3+^ and Fe^2+^ transformation could further enhance enzyme synthesis and activities, promote microbial reproduction and enrichment, and directly and/or indirectly accelerate electron transfer in the denitrification and anammox processes of CWs [53,75,85,86,87]. These further promote more and higher-value patches of relative abundances for these functional genes, which were observed in FeB-HSCW compared with those in the two other systems. Notably, most of them (except for *narG*) were mainly concentrated in the front and middle-upper parts of the system, indicating that Fe-modified biochar could supply more appropriate habitats for N-removing microorganisms and prompt them to use the habitats more differentially (and not exert competitive exclusion). This further suggests that a novel N-removing process, coupled with denitrification, anammox, Feammox (NH_4_^+^-N oxidation with Fe^3+^ reduction), and NAFO (nitrate-dependent anaerobic ferrous oxidizing) in FeB-HSCW, might be the promotor of excellent N removal [88]. Moreover, the relative abundance of *nirS* was higher than those of other functional genes in FeB-HSCW, indicating that Fe-modified biochar was most conducive to *nirS*-denitrifier enrichment. The *nirS*-denitrifier assemblage was an important component in the bacterial community and the primary contributor to efficient N removal in FeB-HSCW [39]. Moreover, Pan et al. [40] suggested that applying biochar to paddy soil could enhance denitrification, which was mainly related to functional gene abundance rather than microbial community structure. However, this study reveals that stronger enhancements of Fe-modified biochar on N removal in CWs are mainly linked to optimized microbial community structures, higher functional gene abundance, and better spatial distribution of N-removing microorganisms.

## 5. Conclusions

To further explore the microbiological mechanism of enhanced N removal in CWs, the influence of Fe-modified biochar on community structures and spatial distributions of N-removing microorganisms in HSCWs was investigated using Illumina MiSeq HTS and q-PCR approaches, respectively. The community structures of *nirS*- and *nirK*-denitrifiers in FeB-HSCW were significantly optimized compared to the two other HSCWs, although no significant difference in their richness and diversity were detected among the HSCWs. Many types of denitrifiers were observed in FeB-HSCW, indicating that Fe-modified biochar could effectively enhance the coexistence of various denitrifying pathways (aerobic and anaerobic, autotrophic, and heterotrophic) for improved N removal. More heterogeneous and complex spatial distributions of denitrifiers and anammox bacteria were observed in FeB-HSCW, according to the relative abundance distributions of *narG*, *napA*, *nirS*, *nirK*, *qnorB*, *cnorB*, *nosZ*-I, *nosZ*-II, and *hzsA*. More and higher-value patches of relative abundances for these functional genes were observed in FeB-HSCW, and most of them were mainly concentrated in the front and middle–upper parts of the system. Thus, Fe-modified biochar can supply more appropriate habitats for N-removing microorganisms to prompt them to use the habitats more differentially while reducing or even eliminating competition exclusion. Therefore, the enhancement by Fe-modified biochar of the microbial N-removal capability in CWs is attributed to optimized microbial community structures, higher functional gene abundance, and better spatial distribution of N-removing microorganisms.

## Figures and Tables

**Table 1:** The richness and diversity of *nirS*- and *nirK*-denitrifier communities in the three constructed wetlands (HSCWs); *n* = 6.

Estimators	*nirS*-Denitrifier			*nirK*-Denitrifier		
	C-HSCW	B-HSCW	FeB-HSCW	C-HSCW	B-HSCW	FeB-HSCW
Reads	94707			101452		
Average Length	392.39			452.089		
OTUs	586	674	795	550	630	802
Sobs	394 ± 76.37	494 ± 103.24	451 ± 49.50	376 ± 21.21	436.5 ± 2.12	444.5 ± 58.69
Shannon	4.58 ± 0.57	4.80 ± 0.54	4.68 ± 0.07	4.60 ± 0.20	4.46 ± 0.31	4.80 ± 0.21
Simpson	0.032 ± 0.016	0.024 ± 0.019	0.022 ± 0.000	0.022± 0.004	0.036 ± 0.019	0.019 ± 0.006
ACE	440.29 ± 35.12	582.12 ± 115.77	523.76 ± 60.54	418.83 ± 48.63	496.53 ± 13.94	487.15 ± 81.17
Chao 1	436.49 ± 44.66	587.13 ± 131.43	523.60 ± 59.32	422.62 ± 61.09	501.58 ± 10.85	480.05 ± 80.34
Coverage	99.19 ± 0.375%	98.51 ± 0.304%	98.74 ± 0.170%	99.27 ± 0.35%	99.00 ± 0.12%	99.21 ± 0.30%

Abbreviations: OTUs, operational taxonomic units; C-HSCW, without biochar and FeB-modified biochar; B-HSCW, with biochar; FeB-HSCW, with FeB-modified biochar.

## Data Availability

No Statements.

## Supplementary Materials

The following are available online at https://www.mdpi.com/1660-4601/18/6/2938/s1. Table S1: The influent characteristics of HSCWs in each operated stage. Table S2: The effluent characteristics of HSCWs in each operated stage. Table S3: Primers for target genes used in q-PCR analysis. Table S4: Protocols and parameters used for q-PCR analysis of the target genes.
