# Supplementary material for: Community Composition and Spatial Distribution of N-Removing Microorganisms Optimized by Fe-Modified Biochar in a Constructed Wetland
Source: Int J Environ Res Public Health. 2021 Mar 13;18(6):2938. doi: 10.3390/ijerph18062938 (PMC8000742; doi:10.3390/ijerph18062938)
Supplement: Supplementary file 1 [file ijerph-18-02938-s001.pdf]

Table S1 The influent characteristics of HSCWs in each operated stage

Parameters	Start-up/Stage Ⅰ	Stage Ⅱ	Stage Ⅲ	Stage Ⅳ	Stage Ⅴ	Stage Ⅵ	Stage Ⅶ
HRT (d)	96	48	24	12	96	48	24
pH	7.07 ± 0.20	7.07 ± 0.20	7.03 ± 0.14	7.06 ± 0.19	7.06 ± 0.10	7.11 ± 0.16	7.00 ± 0.08
DO (mg/L)	3.23 ± 0.08	3.01± 0.20	3.15 ± 0.10	3.21 ± 0.05	3.11 ± 0.08	3.00 ± 0.08	3.12 ± 0.09
ORP (mV)	253.66 ± 16.66	271.36 ± 13.89	256.73 ± 17.05	274.38 ± 5.33	263.64 ± 16.96	249.54 ± 10.09	266.01 ± 14.70
COD (mg/L)	22.580 ± 1.903	23.800 ± 0.700	23.156 ± 0.706	22.800 ± 0.566	22.800 ± 1.145	23.200 ± 0.819	22.844 ± 0.508
TN (mg/L)	9.421 ± 0.238	8.799 ± 0.164	9.123 ± 0.268	10.280 ± 0.117	17.763 ± 0.467	17.695 ± 0.429	17.465 ± 0.392
NH_4^+-N (mg/L)	0.284 ± 0.023	0.279 ± 0.027	0.249 ± 0.020	0.230 ± 0.009	0.569 ± 0.026	0.519 ± 0.022	0.573 ± 0.043
NO_3^--N (mg/L)	7.207 ± 0.420	6.923 ± 0.169	7.001 ± 0.193	7.815 ± 0.155	13.942 ± 0.459	14.015 ± 0.175	13.465 ± 0.383
NO_2^--N (mg/L)	0.008 ± 0.001	0.009 ± 0.001	0.008 ± 0.001	0.010 ± 0.001	0.035 ± 0.002	0.030 ± 0.002	0.028 ± 0.001
C/N ratio	2.4	2.7	2.5	2.2	1.3	1.3	1.3

Table S2 The effluent characteristics of HSCWs in each operated stage

Parameters		TN (mg/L)	NO_3^--N (mg/L)	NH_4^+-N (mg/L)	NO_2^--N (mg/L)	COD (mg/L)	pH	DO (mg/L)	ORP (mV)
Stage Ⅰ	C-HSCW	6.096 ± 0.063	4.128 ± 0.056	0.257 ± 0.018	0.008 ± 0.001	14.840 ± 0.531	6.927 ± 0.074	1.443 ± 0.144	71.39 ± 16.55
	B-HSCW	2.267 ± 0.149	0.813 ± 0.016	0.034 ± 0.009	0.005 ± 0.000	9.759 ± 0.545	7.235 ± 0.040	1.323 ± 0.083	52.80 ± 7.97
	FeB-HSCW	1.253 ± 0.027	0.338 ± 0.013	0.038 ± 0.007	0.002 ± 0.000	8.214 ± 0.516	7.082 ± 0.059	1.242 ± 0.124	39.29 ± 7.38
Stage Ⅱ	C-HSCW	6.509 ± 0.103	4.555 ± 0.093	0.375 ± 0.028	0.015 ± 0.000	19.067 ± 0.346	6.925 ± 0.070	1.133 ± 0.113	-33.37 ± 12.96
	B-HSCW	3.749 ± 0.090	1.826 ± 0.048	0.037 ± 0.009	0.012 ± 0.000	14.289 ± 0.459	7.300 ± 0.059	0.640 ± 0.087	-87.94 ± 8.92
	FeB-HSCW	2.504 ± 0.056	1.364 ± 0.047	0.056 ± 0.011	0.002 ± 0.000	11.911 ± 0.285	7.135 ± 0.083	0.523 ± 0.097	-121.47 ± 7.67
Stage Ⅲ	C-HSCW	8.107 ± 0.171	5.550 ± 0.103	0.404 ± 0.013	0.016 ± 0.000	20.400 ± 0.387	7.007 ± 0.063	1.263 ± 0.143	50.67 ± 16.40
	B-HSCW	5.115 ± 0.180	2.965 ± 0.094	0.056 ± 0.008	0.013 ± 0.000	17.333 ± 0.245	7.283 ± 0.070	0.892 ± 0.112	-29.534 ± 12.93
	FeB-HSCW	4.445 ± 0.090	2.513 ± 0.087	0.108 ± 0.011	0.005 ± 0.000	14.489 ± 0.318	7.137 ± 0.091	0.755 ± 0.092	-76.21 ± 5.54
Stage Ⅳ	C-HSCW	9.877 ± 0.077	7.199 ± 0.085	0.471 ± 0.010	0.021 ± 0.001	20.756 ± 0.573	6.940 ± 0.106	2.172 ± 0.118	189.77 ± 13.61
	B-HSCW	8.960 ± 0.139	6.274 ± 0.071	0.141 ± 0.005	0.019 ± 0.001	18.800 ± 0.400	7.303 ± 0.064	1.745 ± 0.098	88.21 ± 11.32
	FeB-HSCW	7.814 ± 0.128	5.469 ± 0.084	0.193 ± 0.006	0.007 ± 0.000	17.333 ± 0.332	7.133 ± 0.065	1.527 ± 0.078	52.61 ± 9.00

Stage Ⅴ	C-HSCW	12.582 ± 0.309	8.795 ± 0.323	0.505 ± 0.025	0.032 ± 0.001	16.311 ± 0.679	6.940 ± 0.072	1.175 ± 0.105	65.04 ± 12.07
	B-HSCW	5.253 ± 0.121	2.759 ± 0.122	0.090 ± 0.013	0.024 ± 0.001	10.867 ± 0.574	7.245 ± 0.069	1.088 ± 0.063	37.39 ± 9.21
	FeB-HSCW	3.539 ± 0.106	1.770 ± 0.117	0.298 ± 0.018	0.011 ± 0.001	9.067 ± 0.490	7.110 ± 0.069	1.037 ± 0.104	25.74 ± 11.93
Stage Ⅵ	C-HSCW	14.122 ± 0.183	10.275 ± 0.199	0.781 ± 0.028	0.049 ± 0.001	20.689 ± 0.389	6.993 ± 0.054	0.942 ± 0.139	-27.74 ± 5.65
	B-HSCW	8.808 ± 0.112	4.404 ± 0.195	0.118 ± 0.027	0.046 ± 0.001	15.511 ± 0.414	7.283 ± 0.045	0.513 ± 0.109	-93.98 ± 7.66
	FeB-HSCW	6.317 ± 0.077	3.887 ± 0.172	0.304 ± 0.031	0.023 ± 0.003	13.000 ± 0.447	7.138 ± 0.071	0.397 ± 0.094	-126.63 ± 9.48
Stage Ⅶ	C-HSCW	16.451 ± 0.127	11.467 ± 0.223	0.962 ± 0.033	0.055 ± 0.001	21.911 ± 0.722	6.938 ± 0.071	1.172 ± 0.131	41.63 ± 10.32
	B-HSCW	10.905 ± 0.197	6.698 ± 0.143	0.263 ± 0.018	0.050 ± 0.001	18.489 ± 0.226	7.267 ± 0.050	0.833 ± 0.119	-34.92 ± 8.69
	FeB-HSCW	9.426 ± 0.225	5.855 ± 0.209	0.371 ± 0.026	0.024 ± 0.001	15.444 ± 0.343	7.102 ± 0.067	0.743 ± 0.109	-62.56 ± 6.29

Table S3 Primers for Target Genes used in qPCR Analysis

Target genes	Primer	Primer sequence (5′-3′)	Amplification size (bp)	References
bacterial 16S rRNA (*bacteria*)	338F	ACTCCTACGGGAGGCAGCAG	180	Muyzer et al., 1993
	518R	ATTACCGCGGCTGCTGG		
anammox 16S rRNA (*amx*)	AMX809F	GCCGTAAACGATGGGCACT	257	Tsushima et al., 2007
	AMX1066R	AACGTCTCACGACACGAGCTG		
narG	1960m2f	TA(CT)GT(GC)GGGCAGGA(AG)AAACTG	100	Lopez-Gutierrez et al., 2004
	2050m2r	CGTAGAAGAAGCTGGTGCTGTT		
napA	napV67m	AAYATGGCVGARATGCACCC	488	Henry et al., 2008
	napV17m	GRTTRAARCCCATSGTCCA		
nirS	cd3aF	GT(C/G) AAC GT(C/G) AAG GA(A/G) AC(C/G) GG	425	Throback et al., 2004

	R3cd	GA(C/G) TTC GG(A/G) TG(C/G) GTC TTG A		
nirK	F1aCu	ATC ATG GT(C/G) CTG CCG CG	473	Wu et al., 2017
	R3Cu	GCC TCG ATC AG(A/G) TTG TGG TT		
qnorB	qnorB2F	GGNCAYCARGGNTAYGA	262	Braker and Tiedje, 2003
	qnorB5R	ACCCANAGRTGNACNACCCACCA		
cnorB	cnorB2F	GACAAGNNNTACTGGTGGT	389	Braker and Tiedje, 2003
	cnorB6R	GAANCCCCANACNCCNGC		
nosZ-I	nosZ2F	CGCRACGGCAASAAGGTSMSSGT	267	Henry et al., 2006
	nosZ2R	CAKRTGCAKSGCRTGGCAGAA		
nosZ-II	nosZ-II-F	CTIGGICCIYTKCAYAC	720	Jones et al., 2013
	nosZ-II-R	GCIGARCARAAITCBGTRC		
hzsA	hzsA 1597F	WTYGGKTATCARTATGTAG	260	Harhangi et al., 2012
	hzsA 1857R	AAABGGYGAATCATARTGGC		
amoA (AOA)	Arch-amoAF	STAATGGTCTGGCTTAGACG	635	Francis et al., 2005
	Arch-amoAR	GCGGCCATCCATCTGTATGT		
amoA (AOB)	amoA-1F	GGGGTTTCTACTGGTGGT	491	Rotthauwe et al., 1997
	amoA-2R	CCCCTCKGSAAAGCCTTCTTC		
nxrA	F1nxrA	CAGACCGACGTGTGCGAAAG	322	Poly et al., 2008
	R1nxrA	TCYACAAGGAACGGAAGGTC		

Table S4 Protocols and Parameters used for qPCR Analysis of the Target Genes.

Target gene	Programs
bacterial 16S rRNA (*bacteria*)	Pre-denaturation at 98 °C for 2min; 40 cycles of denaturation at 98 °C for 5s, and annealing at 64.5 °C for 30s
anammox 16S rRNA (*amx*)	Pre-denaturation at 98 °C for 2min; 40 cycles of denaturation at 98 °C for 5s, and annealing at 58 °C for 30s
narG	Pre-denaturation at 98 °C for 2min; 40 cycles of denaturation at 98 °C for 5s, annealing at 55 °C for 30s, and extension at 72 °C for 30s
napA	Pre-denaturation at 98 °C for 2min; 40 cycles of denaturation at 98 °C for 5s, and annealing at 61.5 °C for 30s
nirS	Pre-denaturation at 98 °C for 2min; 40 cycles of denaturation at 98 °C for 5s, annealing at 58 °C for 30s, and extension at 72 °C for 30s
nirK	Pre-denaturation at 98 °C for 2min; 40 cycles of denaturation at 98 °C for 5s, annealing at 66.5 °C for 1min, and extension at 72 °C for 1min
qnorB	Pre-denaturation at 98 °C for 2 min; 40 cycles of denaturation at 98 °C for 5s, annealing at 53 °C for 1min, and extension at 72 °C for 1min
cnorB	Pre-denaturation at 98 °C for 2min; 40 cycles of denaturation at 95 °C for 5s, annealing at 56.6 °C for 1min, and extension at 72 °C for 1min
nosZ-I	Pre-denaturation at 98 °C for 2min; 40 cycles of denaturation at 98 °C for 5s, and annealing at 64 °C for

	30s
nosZ-II	Pre-denaturation at 95 °C for 2min; 40 cycles of denaturation at 95 °C for 5s, annealing at 45.8 °C for 1min, and extension at 72 °C for 1min
hzsA	Pre-denaturation at 98 °C for 2min; 40 cycles of denaturation at 98 °C for 5s, annealing at 46 °C for 1min, and extension at 72 °C for 30s
amoA (AOA)	Pre-denaturation at 98 °C for 2min; 40 cycles of denaturation at 98 °C for 5s, annealing at 53.4 °C for 45s, and extension at 72 °C for 45s
amoA (AOB)	Pre-denaturation at 98 °C for 2min; 40 cycles of denaturation at 98 °C for 5s, annealing at 61.5 °C for 1min, and extension at 72 °C for 1min
nxrA	Pre-denaturation at 98 °C for 2min; 40 cycles of denaturation at 98 °C for 5s, annealing at 61.5 °C for 45s, and extension at 72 °C for 30s

References

Braker, G., Tiedje, J.M., 2003. Nitric oxide reductase (norB) genes from pure cultures and environmental samples. Appl. Environ. Microbiol. 69, 3476–3483.

Francis, C.A., Roberts K.J., Beman J.M., Santoro A.E., Oakley B.B., 2005. Ubiquity and diversity of ammonia-oxidizing archaea in water columns and sediments of the ocean. Proc Natl Acad Sci U S A, 102, 14683-14688.

Harhangi, H.R., Le Roy, M., van Alen, T., Hu, B.L., Groen, J., Kartal, B., Tringe, S.G., Quan, Z.X., Jetten, M.S.M., Op den Camp, H.J.M., 2012. Hydrazine synthase, a unique phylomarker with which to study the presence and biodiversity of anammox bacteria. Appl. Environ. Microbiol. 78 (3), 752-758.

Henry, S., Bru, D., Stres, B., Hallet, S., Philippot, L., 2006. Quantitative detection of the *nosZ* Gene, encoding nitrous oxide reductase, and comparison of the abundances of 16S rRNA, *narG*, *nirK*, and *nosZ* Genes in Soils. Appl. Environ. Microbiol. 72(8), 5181-5189.

Henry, S., Texier, S., Hallet, S., Bru, D., Dambreville, C., Chèneby, D., Bizouard, F., Germon, J.C., Philippot, L., 2008. Disentangling the rhizosphere effect on nitrate reducers and denitrifiers: insight into the role of root exudates. Environ. Microbiol. 10, 3082-3092.

Jones, C.M., Graf, D.R.H., Bru, D. Philippot, L., Hallin, S., 2013. The unaccounted yet abundant nitrous oxide-reducing microbial community: a potential nitrous oxide sink. The ISME J. 7, 417-426.

Lopez-Gutierrez, J.C., Henry, S., Hallet, S., Martin-Laurent, F., Catroux, G., Philippot, L., 2004. Quantification of a novel group of nitrate-reducing bacteria in the environment by real-time PCR. J. Microbiol. Methods 57, 399-407.

Muyzer, G., De Waal, E.C. and Uitterlinden, A.G., 1993. Profiling of complex microbial populations by denaturing gradient gel electrophoresis analysis of polymerase chain reaction-amplified genes coding for 16S rRNA. Applied and Environmental Microbiology 59 (3), 695-700.

Poly, F., Wertz, S., Brothier, E., Degrange, V., 2008. First exploration of Nitrobacter diversity in soils by a PCR cloning-sequencing approach targeting functional gene nxrA. FEMS Microbiol. Ecol. 63, 132–140.

Rotthauwe J.H., Witzel K.P., Liesack W., 1997. The ammonia monooxygenase structural gene amoA as a functional marker: molecular fine-scale analysis of natural ammonia-oxidizing populations. Appl. Environ. Microbiol. 63, 4704-4712.

Throback, I.N., Enwall, K., Jarvis, A. and Hallin, S., 2004. Reassessing PCR primers targeting nirS, nirK and nosZ genes for community surveys of denitrifying bacteria with DGGE. Fems Microbiology Ecology 49 (3), 401-417.

Tsushima, I., Kindaichi, T. and Okabe, S., 2007. Quantification of anaerobic ammonium-oxidizing bacteria in enrichment cultures by real-time PCR. Water Research 41 (4), 785-794.

Wu, H.L., Wang, X.Z., He, X.J., Zhang, S.B., Liang, R.B., Shen, J., 2017. Effects of root exudates on denitrifier gene abandance, community structure and activity in a micro-polluted constructed wetland. *Sci. Total Environ.* 598, 697-703.
